# Prokaryotic community structure and respiration during long-term incubations

**DOI:** 10.1002/mbo3.25

**Published:** 2012-06

**Authors:** Federico Baltar, Markus V Lindh, Arkadi Parparov, Tom Berman, Jarone Pinhassi

**Affiliations:** 1 Marine Microbiology, School of Natural Sciences, Linnaeus UniversityBarlastgatan 11, SE-391 82 Kalmar, Sweden; 2 The Yigal Allon Kinneret Limnological Laboratory, Israel Oceanographic and Limnological ResearchIL-14102 Tiberias, Israel

**Keywords:** Bacterioplankton, biological oxygen demand, community composition, incubation, respiration

## Abstract

Despite the importance of incubation assays for studies in microbial ecology that frequently require long confinement times, few reports are available in which changes in the assemblage structure of aquatic prokaryotes were monitored during long-term incubations. We measured rates of dissolved organic carbon degradation and microbial respiration by consumption of dissolved oxygen (DO) in four experiments with Lake Kinneret near-surface water and, concomitantly, we analyzed the variability in prokaryotic community structure during long-term dark bottle incubations. During the first 24 h, there were only minor changes in bacterial community composition. Thereafter there were marked changes in the prokaryotic community structure during the incubations. In contrast, oxygen consumption rates (a proxy for both respiration and dissolved organic carbon degradation rates) remained stable for up to 10–23 days. This study is one of the first to examine closely the phylo-genetic changes that occur in the microbial community of untreated freshwater during long-term (days) incubations in dark, sealed containers. Novel information on the diversity of the main bacterial phylotypes that may be involved in dissolved organic matter degradation in lake Kinneret is also provided. Our results suggest that, under certain ecological settings, constant community metabolic rates can be maintained as a result of shifts in community composition.

## Introduction

Incubation experiments are often used to determine microbial activities in natural water samples. During these incubations, the uptake of different compounds or the changes in the concentration of specific elements are measured. Incubation times differ in length depending on the targeted function. During short-term incubations (hours), such as used for measuring bacterial production (Kirchman et al. 1985), the prokaryotic community composition is assumed to be stable. However, there are many other estimations of key functional parameters that require much longer times, for example, measurements of respiration, rates of dissolved organic carbon degradation, and bacterial growth efficiency (Carlson and Ducklow 1996; Del Giorgio and Cole 1998). In the water industry, since 1908 the determination of bio-logical oxygen demand (BOD), a standard method of gauging the organic load in water, has been based on five-day incubations. During long-term incubations (days); it is feasible that changes in microbial community structure and abundance may occur that could drastically affect the outcome of the measurements—since bacterial populations differ in both metabolic and enzymatic properties (Martinez et al. 1996; Garrity 2001)—leading to results that potentially do not reflect true in situ rates or concentrations.

Long-term incubations (days to weeks) are much longer than prokaryotic division times and therefore raise the question of whether changes in microbial community composition due to confinement alter the rates that are measured (Massana et al. 2001). However, despite the routine utilization of long-term incubations in many aspects of water research and monitoring, it is still unclear if changes in the community structure of aquatic prokaryotes do take place during such incubations and what the consequences thereof may be. Previously obtained results are extremely difficult to compare because these kind of experiments are very sensitive to the physical, chemical, and biological characteristics of the samples tested, and the results can differ significantly with different conditions applied during incubations. Long-term sample incubations can influence final bacterial cell concentrations (Bischofberger et al. 1990), grazing/bacterivory rates (Marrase et al. 1992), and affect bacterial viability/activity parameters (Jürgens et al. 2000) in different aquatic environments. Ferguson et al. (1984) found that the bacterio-plankton community in Onslow Bay (Frying Pan Shoals Tower) changed significantly within 16 h of confinement, from a community dominated by noncultivable to one dominated by cultivable bacteria. In contrast, Lee and Fuhrman (1991) reported only minor changes in confined bacterioplankton from an oligotrophic Pacific station, accounting for at most 15% differences in DNA hybridization between the original water sample and a sample kept in 20-L containers for two days. Massana et al. (2001) found significant differences in both bacterial production and in bacterioplankton phylo-genetic composition at the beginning as compared to the end of long-term incubations (eight to 10 days). Gattuso et al. (2002) also reported changes in respiration rates and in bacterial abundance, cell volume, and assemblage composition during discrete incubations (55 h) of freshwater planktonic communities.

From their results, Massana et al. (2001) concluded that “long-term bottle incubations mostly measure the activity of a few opportunistic bacteria and not that of the original assemblage.” Although few studies have directly addressed the question, Massana et al. (2001) suggested that changes in prokaryotic assemblage composition due to differential outgrowth of specific phylotypes are the principal reason for any alteration of metabolic rates observed during incubations.

However, Comte and del Giorgio (2011), exploring the role that community composition plays in shaping metabolic responses of bacterial communities, recently suggested that community composition influences the pathways of community responses but not the metabolic outcome itself, the latter being determined by the environmental drivers that can be attained through multiple alternative configurations of community composition. Recently, Ferreira and Chauvet (2012) also found a lack of relationship between the identity of the dominant species (of aquatic hyphomycetes) and community performance (here the loss of litter mass), suggesting that microbial assemblages have the capacity to buffer changes in processes due to changes in species dominance. If community metabolic rates are not necessarily changed by shifts in bacterioplankton community structure, these rates could thus still accurately reflect in situ levels.

The aim of this study was to examine the dynamics of oxygen consumption and the variability in bacterio-plankton community composition during long incubations, and to determine the potential links between variability in community respiration (CR) and phylogenetic shifts. We monitored changes in both bacterioplankton phylogenetic composition and a community metabolic rate parameter (oxygen consumption) during long-term (from eight to 23 days) incubations in four experiments (Table 1) using near-surface samples from a central, pelagic site (Station A) in Lake Kinneret, Israel. The rate of dissolved oxygen (DO) consumption reflecting CR is a key community metabolic variable and was especially suitable for our purpose because its determination in terms of dissolved O_2_ did not require any conversion factor or prefiltration step for its determination. The prokaryotic community composition was determined not only at initial and final times as in the studies by Massana et al. (2001) and Gatusso et al. (2002), but at several times during each experiment.

**Table 1:** Summary of the experiments performed, including the dates, temperature (Temperature; °C), chlorophyll concentration (Chl; μg/L), incubation days, dissolved organic carbon (DOC) concentration, daily community respiration (CR) measured in situ over 24 h on Day 0, or as daily average over long-term incubations, and significance (*P*-value) of the linear and exponential regression models. eCR, CR calculated from the exponential fit equation. Note that in Experiment 4, the last day was not included in the calculation of CR_fin_ (see main text for details)

Experiment	Date	Temperature	Chl	Incubation days	DOC (mg/L)	CR_24_^1^ (mg/L/day)	CR_fin_^2^ (mg/L/day)	eCR_24_^1^ (mg/L/day)	eCR_fin_^2^ (mg/L/day)	*P*-value linear (mg/L/day)	*P*-value exponential (mg/L/day)
1	27 November 2003	23.5	6.8	23	3.42	0.090	0.091	0.100	0.090	<0.0001	<0.0001
2	14 March 2004	17.1	73.2^3^	8	5.77	0.759	0.441	0.501	0.430	0.0010	0.0024
3	22 June 2004	25.5	16.5	12	4.80	0.401	0.461	0.210	0.182	0.0069	0.0043
4	24 October 2004	26.7	9.0	16	4.88	0.183	0.181	0.152	0.140	0.0018	0.0014

1 Based on 24-h incubations on Day 0.

2 Calculated as: (DO_Last Day_– DO_Day0_)/days incubation.

3 Dense *Peridinium* bloom.

In these experiments, we intentionally extended the “classical” five-day BOD incubation time so that we could check how long the measured respiration rates in the BOD bottles remained similar to those measured initially. Additionally, the longer incubation times allowed us to follow changes in the specific phylotypes within the microbial community that occurred at different incubation stages, possibly indicating phylotypes that responded, or contributed, to changes in the quality and/or quantity of organic matter.

## Materials and Methods

Samples were taken from near surface (1-m depth), at a central, pelagic site (Station A) in Lake Kinneret. Planktonic CR was measured in triplicate lake water samples in dark BOD bottles that were incubated at in situ conditions between 10 and 23 days (Table 1). Potentiometric titrations (azide modification of the Winkler method) with a high precision (±2.0 μL) 719S Metrohm Titrino titrator (Metrohm Ltd., Herisau, Switzerland) were made to determine oxygen concentrations (Berman et al. 2010). Dissolved organic carbon (DOC) was measured in GF/F filtered water using an Oceanography International Analytical (OI Analytical Inc., College Station, Texas, USA) Total Organic Carbon Analyzer (TOC). Chlorophyll-a was measured fluoro-metrically (Holm-Hansen et al. 1965). Microbial biomass from BOD bottles for DNA extraction (incubated in parallel to those used for BOD measurements) was obtained by each sampling day filtering approximately 400 mL of lake water from the incubation bottles (pooled samples from duplicate bottles) onto separate 47-mm diameter and 0.2-μm pore size polycarbonate filters (Supor-200, PALL Life Sciences, Ann Arbor, MI, USA) at <200 mmHg. Filters were stored frozen at –70°C in sucrose buffer; community DNA was obtained using a standard phenol-extraction protocol (extractions were done in 2-mL eppendorf tubes) (Schauer et al. 2003). Denaturing gradient gel electrophoresis (DGGE) analyses were performed as previously described (Schauer et al. 2003). Briefly, a 16S rRNA gene fragment (approximately 550-bp long) was amplified by PCR, using the bacterium-specific primer 358f (5′-CGCCCGCCGCGCGCGGCGGGCGGGGCGGGGGCACG GGGGGCCTACGGGAGGCAGCAG) that is complementary to positions 341–358 (*Escherichia coli* numbering) and has a GC clamp (underlined) and the universal primer 907rm [5′-CCGTCAATTC(A/C)TTTGAGTTT] that is complementary to positions 927–907. PCR products were loaded on a 6% polyacrylamide gel with a DNA-denaturant gradient ranging from 40% to 80%. The gel was run at 100 V for 16 h at 60°C in 1× TAE running buffer. DGGE bands were excised, reamplified, and verified by a second DGGE. Bands were sequenced using primer 358f without the GC-clamp, with the BigDye terminator cycle-sequencing kit and an ABI PRISM model 377 (v3.3) automated sequencer (Applied Biosystems Division of Perkin Elmer Corporation, Foster City, CA, USA). A matrix was constructed for all lanes taking into account the relative contribution of each band (%) to the total intensity of the lane of the DGGE gel images. DGGE fingerprints were used to construct dendrograms using the package Vegan in R 2.12.0 (Oksanen et al. 2011), applying the Lance-Williams coefficients, euclidean distances, and unweighted-pair group means analysis (UPGMA). Based on this matrix, we obtained a dendrogram based on UPGMA clustering (Euclidean distances) (Statistica 6.0, Statsoft Inc., Tulsa, USA). Our 16S rRNA gene sequences were compared to sequences in GenBank using BLAST. Sequences have been deposited in GenBank under the accession numbers JQ937365–JQ937380.

## Results and Discussion

**Figure 1:**
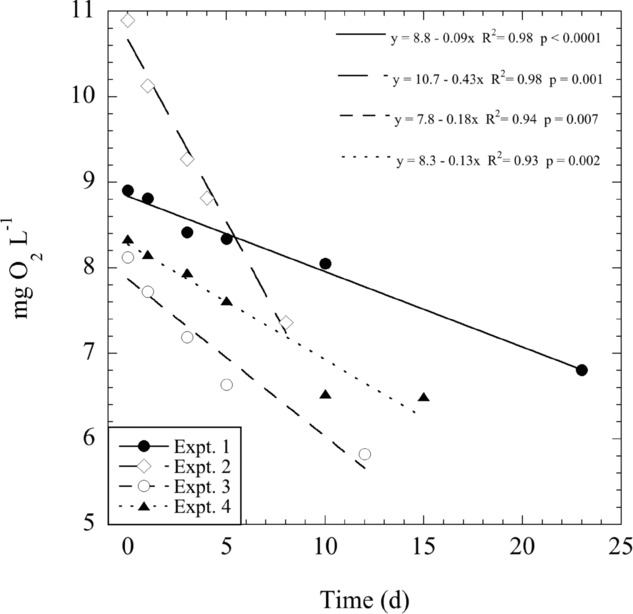
Decrease in oxygen concentration (mg O_2_ L^−1^) during four different experiments. A linear regression model was applied to each of the experiments and the equations added to the plot. Standard deviations are not visible since they are smaller than the symbols.

Lake Kinneret is a warm monomictic lake with a surface area of 170 km^2^, and mean and maximum depths of 24 and 43 m, respectively. Homothermy occurs between late December and early March, with minimum water temperatures usually more than 14°C. The lake is strongly stratified from about April to December, with maximum epilimnetic temperatures reaching 29–30°C. Bacterial respiration has been estimated on average as approximately 50% of CR (Berman et al. 2010) in this lake. During our study, CR rates differed depending on the sampling season (Fig. 1), with lowest values in autumn of both years (Experiments 1 and 4), probably related to the very different initial levels of ambient phytoplankton in the four experiments (as indicated by the chlorophyll concentrations in Table 1). The highest CR rates were measured during March 2004 (Experiment 2), which was run when there was an intense bloom of the dinoflagellate *Peridinium gatunense* in the lake, coinciding with the highest chlorophyll and DOC concentrations of all experiments (Table 1). These results are consistent with CR data measured from 2001 to 2007, which consistently showed maximum rates during the period from February to May (Berman et al. 2010). A close relationship between chlorophyll concentrations and biological oxygen demand (BOD) has been reported for several aquatic ecosystems (Zsolnay 1975; Fallon and Brock 1979; Jonas and Tuttle 1990). Extremely high BOD during *Peridinium* blooms in 2001 and 2006 in this lake was also noted by Ostapenia et al. (2009). Berman et al. (2004) also found that BOD measured over five days was significantly correlated to prokaryotic production and CR. They suggested that BOD determinations made over five or more days could serve as a good measure both of community metabolic rates and of the availability of labile organic matter in aquatic systems. It is particularly remarkable to find that, in our experiments, the oxygen concentration followed a linear decrease, not only for five days, but also up to eight and even 23 days (Fig. 1).

The highly significant linearity (linear or exponential models for the regression were similarly low and statistically significant; see Table 1) of dissolved O_2_ consumption observed in these experiments demonstrates the constancy of the CR rate measurement over relatively long periods. The exception to linearity of the last sample point in Experiment 4 may have been caused by bacterial depletion of the available organic matter. At the beginning of each experiment, CR rates in the same water samples were also determined independently by measuring the dissolved O_2_ consumption over 24 h in dark bottles suspended in situ. (Note this was the standard method used for routine CR measurements.) The daily respiration rate when calculated from the long-term incubations in Experiments 1, 3, and 4 (until day 10 only) was almost identical to that measured directly in situ (Table 1). The same was found when the CR was calculated from the equation of the exponential model (Table 1), suggesting that, independently of the type of regression model, the CRs from the long-term incubations were similar to those determined directly in situ. In Experiment 2, carried out during the extreme conditions of a dense *P. gatunense* bloom, CR measured in situ over 24 h was 1.7-fold higher than that computed from the long-term (16 days) incubation (Table 1). Despite the very limited number of experiments, these results suggest that initial CR rates (measured over 24 h) may be close to CR rates obtained during long-term incubations as long as organic matter is available for the metabolism of the enclosed microbial community.

**Figure 2:**
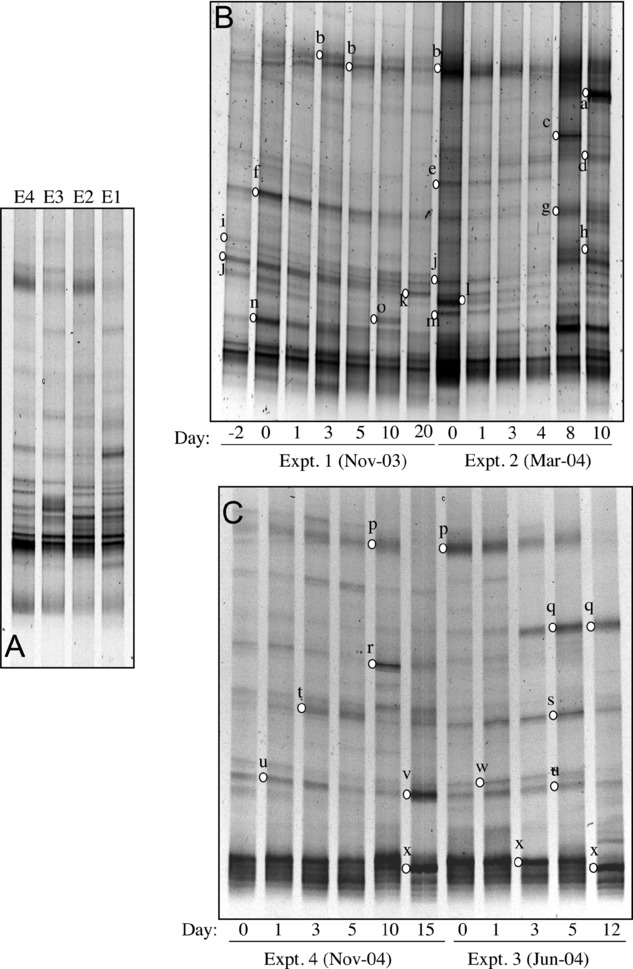
DGGE fingerprints of bacterial assemblages visualized by DGGE of PCR-amplified partial 16S rRNA genes from the initial time zero samples of the four experiments (A), during experiments 1 and 2 (B), and 3 and 4 (C).

**Figure 3:**
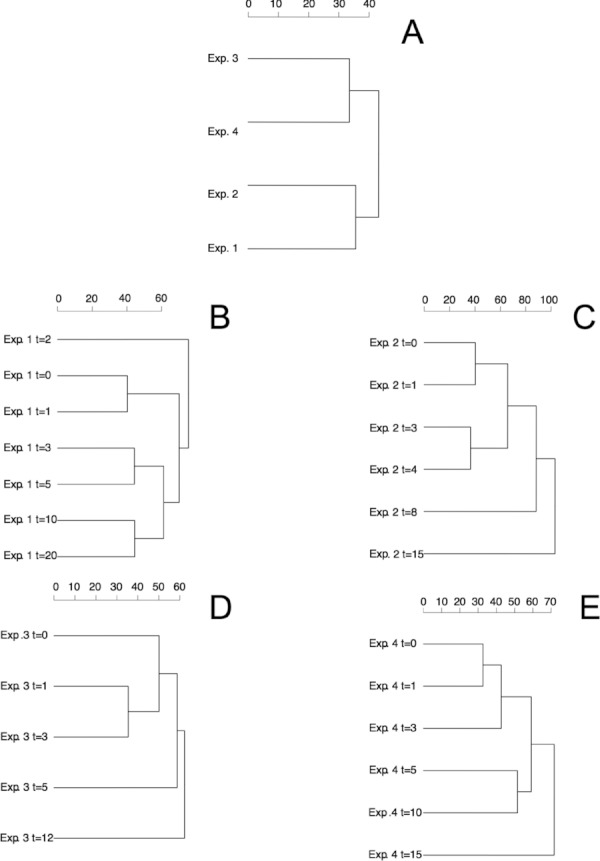
Dendrogram obtained by analysis of the DGGE fingerprints by Euclidean distances comparing the initial samples from the four experiments (A), and during experiment 1 (B), experiment 2 (C), experiment 3 (D), and experiment 4 (E). Note that one sample taken in the same sampling station but two days before starting the experiment 1 (B) is also included (*t* = –2).

**Figure 4:**
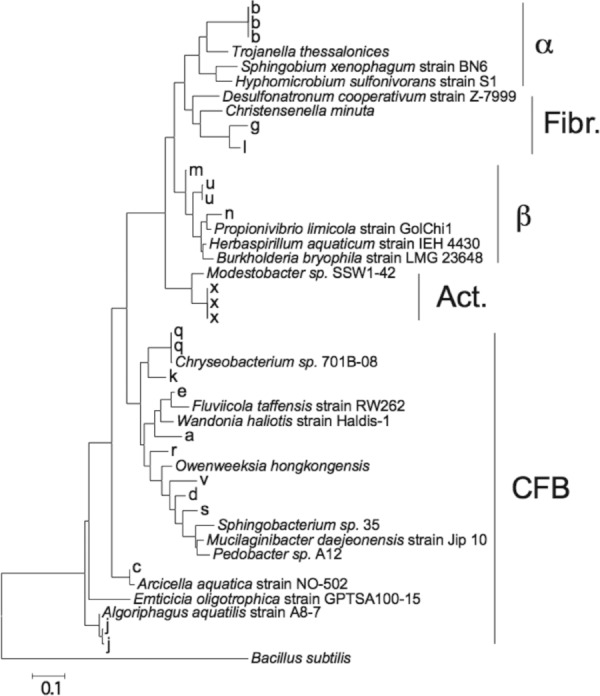
Phylogenetic tree depicting relationships among partial 16S rRNA gene sequences of bacterial phylotypes detected during the four experiments (shown in letters according to Table 2) compared to type species of representative genera in the different phylums. Scale bar depicts 0.1 substitution per nucleotide position. Fibr., Fibrobacteres; Act., Actinobacteria; CFB, Bacteroidetes.

To date there are no published works where the surface bacterioplankton community composition of Lake Kinneret has been reported. In our study, the initial prokaryotic phylogenetic composition (as determined by DGGE) of the natural community in the lake was different depending on the sampling period (Figs. 2A and 3A), as occurred for the CR rates (Fig. 1) and the DOC concentrations (Table 1). Albeit community structure studies using next generation massive sequencing are increasingly common, fingerprinting techniques such as the DGGE approach used in this study, are highly adequate for determining the responses of dominant phylotypes; recently Pommier et al. (2010) showed that community analysis using all the data from 454 sequencing provides the same result as using only data from the 30 most abundant operational taxonomic units (OTUs) (i.e., the same resolution of DGGE). Although it is difficult to decipher a seasonal pattern of specific phylotypes from our limited data set, it is possible to see that some phylotypes were present at the initial time in all experiments despite the strong variation found in DOC concentrations throughout the year. This was the case for the Actinobacteria (band x) and the SAR11 freshwater cluster (Alphaproteobacteria; bands b, p) (Figs. 2 and 4 Table 2); Actinobacteria made up a large portion of the bacterial communities both in the initial water samples and during the experiments consistent with reports that they typically represent between 30% and 70% of total bacteria in freshwater habitats (Glöckner et al. 2000; Sekar et al. 2003; Allgaier and Grossart 2006). The persistency of Actinobacteria throughout our study could be related to the different phylogenetic lineages of freshwater Actino-bacteria reported previously (Zwart et al. 2002; Warnecke et al. 2004) and not only to a single phylotype. Actinobacteria lineages seem to cover different ecological niches showing great ecophysiological plasticity, with subgroups constituting different ecotypes (Hahn and Pöckel 2005; Allgaier et al. 2007). For example, Holmfeldt et al. (2009) found that different Actinobacteria subclusters responded to contrasting environmental parameters along environmental gradients in the brackish northern Baltic Sea. The aquatic bacterial group SAR11 cluster is also formed by several subgroups, which in some cases seem to be correlated with specific environments (Field et al. 1997; Brown and Fuhrman 2005; Kan et al. 2008). Therefore, eco-physiological plasticity based on a significant number of lineages seems to be an important strategy to overcome seasonal changes in the organic carbon availability in this lake.

**Table 2:** Phylogenetic affiliation of 16S rRNA gene sequences from excised DGGE bands obtained during the incubation experiments. For each phylotype, closest relative GenBank and closes cultured relative are shown together with the sequence similarity (%) and accession number

Band	Closest cultured relative in Genbank	Percentage	Accession no.	Closest relative in GenBank	Percentage	Accession no.	Class	Phylum
x	*Modestobacter* sp. SSW1–42	91	FM995613.1	Uncultured bacterium clone THBP.0912.68	99	HQ905025.1	Actinobacteria	Actinobacteria
g	*Christensenella minuta*	81	AB490809.1	Uncultured *Fibrobacter* sp. clone hsh-8–12	99	GU323642.1	Fibrobacteres	Fibrobacteres
l	*Desulfonatronum cooperativum* strain Z-7999	81	AY725424.1	Uncultured bacterium clone WR124	100	HM208490.1	Fibrobacteres	Fibrobacteres
b	*Candidatus Odyssella thessalonicensis* L13	85	AF069496.1	Uncultured SAR11 cluster alphaproteobact clone YL221	100	HM856580.1	Alphaproteobacteria	Proteobacteria
o	*Sphingobium xenophagum* strain BN6	84	NR_026304.1	Uncultured *Sphingomonas* sp.	86	AM934758.1	Alphaproteobacteria	Proteobacteria
p	*Hyphomicrobium sulfonivorans* strain S1	81	NR_025082.1	Uncultured SAR11 cluster alphaproteobact clone YL221	97	HM856580.1	Alphaproteobacteria	Proteobacteria
m	*Herbaspirillum aquaticum* strain IEH 4430	90	FJ267649.1	Uncultured proteobacterium clone 2G65	92	GU074082.1	Betaproteobacteria	Proteobacteria
n	*Propionivibrio limicola* strain GolChi1	93	NR_025455.1	Uncultured bacterium clone KZNMV-30-B39	99	FJ712609.1	Betaproteobacteria	Proteobacteria
u	*Burkholderia bryophila*	94	AM489500.1	Uncultured proteobacterium clone 2K44	100	GU074239.1	Betaproteobacteria	Proteobacteria
a	*Cryomorphaceae bacterium Haldis-1*	90	FJ424814.1	Uncultured bacterium	99	FM201105.1	Flavobacteriia	Bacteroidetes
e	*Fluviicola taffensis* strain RW262	92	AF493694.2	Uncultured *Fluviicola* sp. clone Jab PL1W2D1	99	HM486207.1	Flavobacteriia	Bacteroidetes
q	*Chryseobacterium* sp.	99	FN674441.1	*Chryseobacterium* sp. PanRB005	99	AB581571.1	Flavobacteriia	Bacteroidetes
r	*Chryseobacterium* sp.	88	FN674441.1	Uncultured *Bacteroidetes bacterium* clone MEf05b11A2	98	FJ828083.1	Flavobacteriia	Bacteroidetes
i	*Owenweeksia hongkongensis*	82	AB125062.1	Uncultured bacterium clone DP7.4.20	87	FJ612274.1		Bacteroidetes
k	*Owenweeksia hongkongensis*	87	AB125062.1	Unidentified bacterium clone K2–30-6	96	AY344418.1	Flavobacteriia	Bacteroidetes
v	*Owenweeksia hongkongensis*	89	AB125062.1	Uncultured bacterium	99	AB231428.1	Flavobacteriia	Bacteroidetes
c	*Arcicella aquatica* strain NO-502	99	NR_029000.1	*Arcicella* sp. NSW-5	99	HM357635.1	Cytophagia	Bacteroidetes
f	*Sphingobacterium* sp. 35	84	EU595360.1	Uncultured *Bacteroidetes bacterium* clone 2K51	94	GU074246.1		Bacteroidetes
h	*Emticicia oligotrophica* strain GPTSA100–15	91	AY904352.2	Uncultured *Emticicia* sp. clone cuticle_12	93	HQ111160.1	Cytophagia	Bacteroidetes
j	*Algoriphagus aquatilis* strain A8–7	99	EU313811.1	Uncultured *Hongiella* sp. clone XZNMC13	99	U703231.1	Cytophagia	Bacteroidetes
d	*Mucilaginibacter daejeonensis*	91	AB267717.1	Uncultured *Bacteroidetes bacterium* clone CL1H6	99	FJ916256.1	Sphingobacteria	Bacteroidetes
s	*Sphingobacterium* sp. 35	88	EU595360.1	Uncultured bacterium clone DP10.3.3	99	FJ612364.1	Sphingobacteria	Bacteroidetes
t	*Pedobacter* sp. A12	81	HM051286.1	Uncultured *Haliscomenobacter* sp. clone WR41	87	HM208523.1	Sphingobacteria	Bacteroidetes
w	*Pedobacter lentus* strain DS-40	85	EF446146	Uncultured *Bacteroidetes bacterium* clone TK-NE8	95	DQ463716.2	Sphingobacteria	Bacteroidetes

In contrast to the linearity found in the CR rates, marked shifts occurred in the prokaryotic community composition throughout the incubations in all the experiments (Figs. 2 and 3). No abrupt changes were found in prokaryotic community composition during the first 24 h but pronounced shifts were encountered thereafter. The dendrograms derived from the results in all experiments, shown in Fig. 3, illustrate the continuous population changes due to both relative abundance and the appearance or disappearance of bacterial phylotypes. However, we note that although the prokaryotic community composition changed during confinement in all our experiments (Figs. 2B and C, 3B and E), the degree of variability found over the 23 days of incubation of Experiment 1 (Fig. 3B) was much lower than the variability in community composition in a sample from the original water taken just 2 days before the experiment (Days −2 and 0 in Fig. 3). Although this finding could also have been due to changes caused by water mass transport, nevertheless it emphasizes the dynamic nature of the natural system, where shifts in the community composition are continuously occurring. The latter part of Experiment 4 (from Day 10 to 16) is another good example of the decoupling found between community shifts and respiration rates. In this case, no apparent consumption of DO occurred (Fig. 1), but nevertheless we observed changes in the composition of the prokaryotic community. In general, in all these experiments, some phylotypes remained constant, few disappeared, and few appeared toward the end of the incubations. Some phylotypes that were absent initially bloomed (i.e., appeared and disappeared) during the same experiment (Fig. 2).

Phylotypes that were constantly present throughout all the incubations included a member of the SAR11 freshwater cluster (Alphaproteobacteria; band b), five *Bacteriodetes* (bands d, f, i, s, t; including four Sphingobacteria), one *Fibrobacter* (band l), three Betaproteobacteria (bands m, n, u), and one Actinobacteria (band x) (Table 2; Fig. 4). Phylotypes that disappeared during the incubations included two Flavobacteria (bands e and j), and one Alphaproteobacteria (band p). In contrast, phylotypes that appeared toward the end of the incubations (i.e., were favored by the confinement) included four Flavobacteria (bands a, k, q, v; one closely related to *Chryseobacterium* sp.), two *Cythophaga* (bands c, h; one closely related to *Arcicella sp*.), and an uncultured *Fibrobacter* sp. (band g). Finally, phylotypes that bloomed temporarily included one Alphaproteobacteria related to *Sphingomonas* sp. (band o) and a Flavobacteria (band r) (Table 2; Fig. 4).

The genus *Arcicella* was described by Nikitin et al. (2004) to include slowly growing bacteria from a freshwater neuston film. The recently described species *Arcicella rosea* (Kampfer et al. 2009) was shown to require nutrient-poor conditions and was not able to grow on nutrient-rich media such as nutrient agar or tryptone soy agar. A common feature of the genus *Fibrobacter* is that all cultured strains assigned to the genus thus far are capable of cellulose hydrolysis (Amann et al. 1992), suggesting a key role for *Fibrobacter* spp. in cellulose hydrolysis in the environment (McDonald et al. 2009). *Sphingomonas* species have been found to be dominant in bacterioplankton in the brackish Baltic Sea when nutrient availability limited bacterial growth (i.e., under stratified summer conditions; Pinhassi and Hagstrom 2000). Collectively, the development of *Arcicella*, *Fibrobacter,* and *Sphingomonas* populations in our experiments appears likely to be consequence of the progressively lower lability and/or availability of organic matter expected during incubation rather than of a confinement-favored opportunistic behavior.

Throughout the experiments we observed that, although there were shifts in the prokaryotic community during these experiments, there was no predominant growth of only copiotrophic or opportunistic bacteria that appeared and persisted until the end of the incubations. As shown in our experiments, the bottle-enclosed system is dynamic, with the possible development of slow-growing phylotypes (e.g., *Arcicella* sp.) and not only opportunistic ones. Even some of the phylotypes that appeared intermittently were also related to typically slow growers (e.g., *Sphingomonas*) and not to opportunistic taxa as might be expected.

If community composition changes substantially but metabolic rates remain roughly similar, the case could be made that composition does not really matter; if composition does not change but rates do, then one could make the case that resources matter. In our study, despite the marked shifts of bacterial populations observed that might have been expected to affect CR rates, this apparently did not occur as long as there were readily available organic substrates for bacteria. The time after which organic substrates are depleted may differ from one sample to another, so it is not possible to set a fixed length for the reliability of long-term incubations. This study is one of the first to examine closely the phylogenetic changes that occur in a microbial population of untreated freshwater during long-term (days) incubations in dark, sealed containers. Our results suggest that long-term incubations may yield valid results in measurements of certain community metabolic parameters (e.g. CR, bacterial growth efficiencies, rates of dissolved organic carbon degradation) despite shifts in the composition of the prokaryotic community during the course of the incubation. Related to this, Ferreira and Chauvet (2012) suggested that aquatic hyphomycete assemblages have the capacity to buffer changes in processes due to changes in species dominance. Furthermore, Comte and del Giorgio (2011) recently concluded that community composition influences the pathway involved but not the outcome of the metabolic response of bacterioplankton communities to resource shifts. Our results fit well with this proposed scenario, suggesting that, under certain ecological settings, constant community metabolic rates can be maintained as a result of shifts in community composition.
